# Chemical Composition and Antimicrobial Potential of Essential Oil of *Acritopappus confertus* (Gardner) R.M.King & H.Rob. (Asteraceae)

**DOI:** 10.3390/ph15101275

**Published:** 2022-10-17

**Authors:** Rafael Pereira da Cruz, José Walber Gonçalves Castro, Débora Odília Duarte Leite, Natália Kelly Gomes de Carvalho, José Weverton Almeida-Bezerra, Raimundo Luiz Silva Pereira, Fázia Fernandes Galvão Rodrigues, José Jailson Lima Bezerra, Adrielle Rodrigues Costa, Edna Mori, Pablo Antonio Maia de Farias, Henrique Douglas Melo Coutinho, Maria Flaviana Bezerra Morais-Braga, Marcello Iriti, José Galberto Martins da Costa, Fabíola Fernandes Galvão Rodrigues

**Affiliations:** 1 Department of Biological Sciences, Regional University of Cariri, Crato 63105-000, Brazil; 2 Northeast Biotechnology Network, Postgraduate Program in Biotechnology, State University of Ceará, Fortaleza 60740-000, Brazil; 3 Department of Botany, Federal University of Pernambuco, Recife 50670-901, Brazil; 4 Department of Biochemistry, Federal University of Cariri, Barbalha 63180-000, Brazil; 5 CECAPE College, Av. Padre Cícero, Juazeiro do Norte 63024-015, Brazil; 6 Department of Biological Chemistry, Regional University of Cariri, Crato 63105-000, Brazil; 7 Department of Biomedical, Surgical and Dental Sciences, Università degli Studi di Milano, via G. Celoria 2, 20133 Milan, Italy

**Keywords:** antimicrobial, antibiotics, GC/MS, terpenes, myrcene

## Abstract

Microbial resistance has become a worrying problem in recent decades after the abusive use of antibiotics causing the selection of resistant microorganisms. In order to circumvent such resistance, researchers have invested efforts in the search for promising natural substances, such as essential oils. Thus, the objective of this work was to determine the chemical composition of the essential oil of *Acritopappus confertus* leaves, to evaluate its intrinsic effect and its effects in combination with drugs against pathogenic fungi and bacteria, in addition to verifying the inhibition of virulence in *Candida* strains. To this end, the oil was verified by gas chromatography coupled with mass spectrometry (GC/MS). *Candida* strains were used for antifungal assays by means of the serial microdilution technique, in order to determine the average inhibitory concentration (IC_50_), and for the modification assays, sub-inhibitory concentrations (MIC/8) were used. Finally, the natural product’s ability to inhibit the formation of filamentous structures was evaluated. In antibacterial tests, the MIC of the oil against strains of *Staphylococcus aureus* and *Escherichia coli* and its modifying effects in association with gentamicin, erythromycin, and norfloxacin were determined. The major constituent of the essential oil was the monoterpene myrcene (54.71%). The results show that the essential oil has an antifungal effect, with *C. albicans* strains being the most susceptible. Furthermore, the oil can potentiate the effect of fluconazole against strains of *C. tropicalis* and *C. albicans.* Regarding its effect on micromorphology, the oil was also able to inhibit the filaments in all strains. In combination with antibiotics, the oil potentiated the drug’s action by reducing the MIC against *E. coli* and *S. aureus*. It can be concluded that the essential oil of *A. confertus* has potential against pathogenic fungi and bacteria, making it a target for the development of an antimicrobial drug.

## 1. Introduction

Microbial resistance has become one of the most serious public health concerns around the world, considered a major clinical challenge in the treatment of invasive infections. This issue has intensified due to the indiscriminate use of antibiotics and synthetic drugs, increasingly favoring microbial resistance [1,2,3,4]. The survival and spread of resistant microorganisms have been motivators for research aiming to find safe and effective alternatives, or ways to potentiate the effects of current antimicrobial agents [1,5].

The Center for Disease Control and Prevention has declared the rapid growth of antibiotic resistance as a top global concern and estimates that more than 700,000 people die worldwide from diseases caused by resistant strains [6,7]. *Staphylococcus aureus* and *Escherichia coli* are examples of bacteria that cause nosocomial infections that are resistant to available standard drugs, in addition to having a high capacity for pathogenicity and virulence, being associated with severe clinical cases [8,9,10].

Within this problem, fungi of clinical interest also stand out, as representatives of the *Candida* genus can cause issues ranging from superficial and cutaneous infections (dermatomycoses) to more serious problems, such as candidiasis (invasive and disseminated infections) [11]. The best-known species of the genus, *Candida albicans*, is responsible for 90% of cases of fungal infections [11,12,13]. Others, such as *C. tropicalis* and *C. krusei*, also stand out as agents associated with emerging infections, and may present more resistance than strains of *C. albicans*, making the search for new antifungal drugs a necessary alternative [14].

These pathogenic fungi can change their morphology from budding ovoid yeast to filamentous growth (elongated hyphae) in response to different environmental signals, e.g., lack of nitrogen [14,15,16]. This factor can provide virulence characteristics, initially through adhesion, driven by the expression of numerous surface adhesin proteins [17]. Such virulence allows fungi to colonize tissues and express drug resistance [18]. Literature data demonstrate that the virulence of fungi can be ameliorated or eliminated if the morphogenic capacity is compromised [18].

The search for substances that aim to inhibit fungal virulence phenotypes has become constant [19]. In this context, research into essential oils from medicinal plants has grown significantly as a new strategy to control fungal growth and intensify the effects of pharmaceutical drugs against *Candida* strains [20,21,22,23,24].

Essential oils from species of the Asteraceae family have gained prominence in several studies with different purposes, demonstrating impressive microbiological effects [25,26]. This botanical family holds many species with medicinal properties, although only 15% of the species belonging to this family have been studied [25]. It is noteworthy that several classes of secondary metabolites with antimicrobial potential are present in the essential oils of the species.

As a representative of this family with promising properties, *Acritopappus confertus* (Gardner) R.M.King & H.Rob. stands out, popularly known as “cura-facada”, it is used in traditional medicine for skin wounds due to its healing effects [27]. Its chemical composition reports the predominant presence of monoterpenes in its essential oil [28], and these bioactive components may have different modes of action on antimicrobial activity [11].

In this context, it is hypothesized that the essential oil of *A. confertus* can promote antimicrobial effects against different strains of pathogenic fungi and bacteria. In order to achieve such results, the objective of this work was to evaluate the chemical composition of the essential oil of *A. confertus*, as well as to verify its antimicrobial potential and as a modifier of synthetic drugs, in addition to verifying the inhibition of hyphae and pseudohyphae in *Candida* strains.

## 2. Results

### 2.1. Chemical Composition

According to the phytochemical analysis of the essential oil of *A. confertus*, it was possible to identify 10 chemical constituents corresponding to 96.33% of the total composition. The identified compounds belong to the monoterpene and sesquiterpene classes. Myrcene (54.71%), a monoterpene of pharmacological importance, stood out for being the major constituent of the analyzed sample. The constituents β-pinene (18.2%), limonene (6.52%), and β-eudesmol (5.72%) also showed reasonable concentrations. Regarding the phytochemicals with the lowest concentration traces (<1%), α-humulene (0.80%), α-eudesmol (0.21%), and sabinene (0.19%) were identified (Table 1).

### 2.2. Antifungal and Fluconazole Modifying Activity

**Figure 1:**
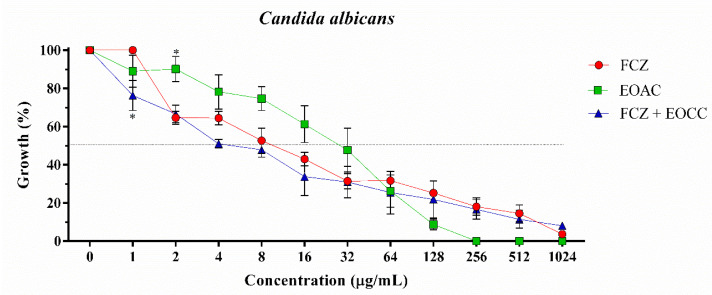
Cell viability curve of *Candida albicans* INCQS 90028 against essential oil of *Acritopappus confertus* (EOAC) and fluconazole (FCZ) in μg/mL. Statistically significant value with * *p* < 0.05.

**Figure 2:**
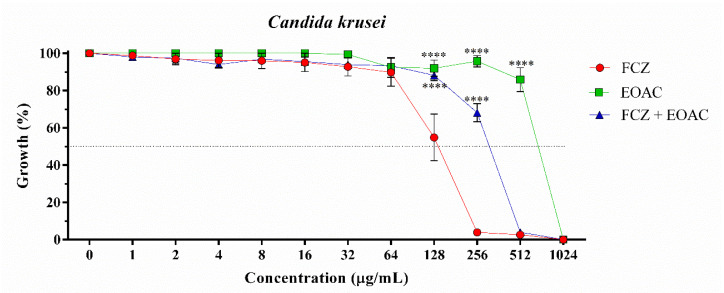
Cell viability curve of *Candida krusei* INCQS 40095 against essential oil of *Acritopappus confertus* (EOAC) and fluconazole (FCZ) in μg/mL. Statistically significant value with **** *p* < 0.0001.

**Figure 3:**
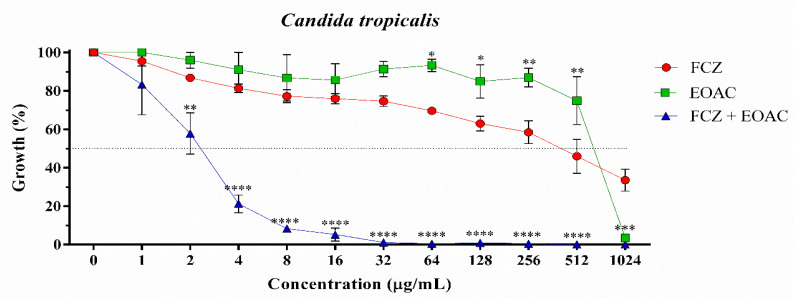
Cell viability curve of *Candida tropicalis* INCQS 40042 against essential oil of *Acritopappus confertus* (EOAC) and fluconazole (FCZ) in μg/mL. Anti-*Candida* potential of essential oil of *Acritopappus confertus* against strains of *Candida tropicalis*. Statistically significant value with * *p* < 0.05, ** *p* < 0.01, *** *p* < 0.001 and **** *p* < 0.0001.

*A. confertus* essential oil showed antifungal activity against *C. albicans* (IC_50_ = 22.19 μg/mL) at a concentration similar to the positive control fluconazole (IC_50_ = 12.33 μg/mL), a standard drug widely used in fungal infections (Table 2) (Figure 1). However, against *C. krusei* and *C. tropicalis* strains, the essential oil showed a low antifungal effect, with IC_50_ values of 592.6 μg/mL and 615.4 μg/mL, respectively (Table 2) (Figure 2 and Figure 3). When associated with fluconazole, the essential oil of *A. confertus* potentiated the antifungal effect against *C. albicans* and *C. tropicalis*, presenting IC_50_ values of 6.53 μg/mL and 2.25 μg/mL, respectively. Based on the behavior of the curve through non-linear regression, the IC_50_ values of the essential oil of the studied plant species were clinically relevant when evaluated alone for *C. albicans*. Regarding the *C. krusei* strain, no positive results were observed for the aforementioned oil tested alone or in association with fluconazole. In addition, the natural product presented a MIC of 256 μg/mL for *C. albicans* (Figure 1), making it a great candidate for the development of new drugs.

### 2.3. Anti-Virulence Potential

**Figure 4:**
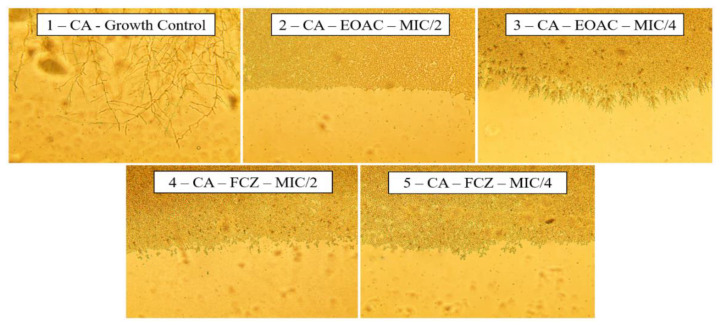
Effects of *Acritopappus confertus* essential oil on the morphological aspects of *Candida albicans*. Slide (S1): Growth control; (S2): Effect of essential oil at a concentration of 128 µg/mL; (S3): Effect of essential oil at a concentration of 64 µg/mL; (S4): Effect of fluconazole at a concentration of 1024 µg/mL; (S5): Effect of fluconazole at a concentration of 512 µg/mL. Display 400× enlarged.

The analysis of the effect of the essential oil of *A. confertus* on the morphology of the yeasts of *C. albicans* was carried out through the depletion of the culture medium, stimulating the yeast to seek nutrients through the emission of hyphae and pseudohyphae. To control the growth of *C. albicans* (Figure 4, Slide 1), it is possible to verify the formation of several hyphae, while when treated with the essential oil of *A. confertus*, there is a significant decrease in the projection of the hyphae, mainly at the concentration of 128 µg/mL (S2), which totally inhibited filament formation.

**Figure 5:**
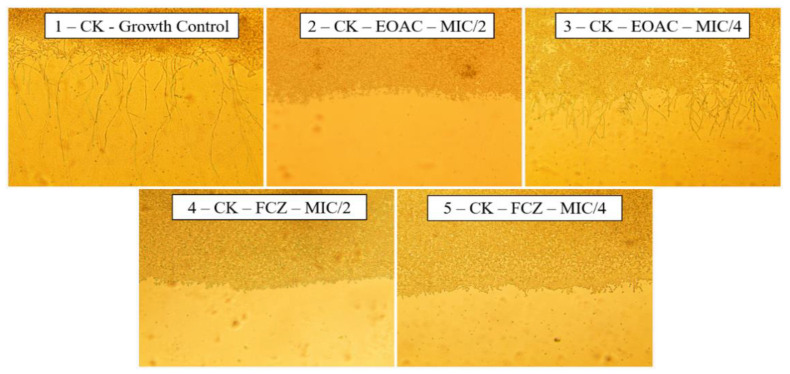
Effects of *Acritopappus confertus* essential oil on the morphological aspects of *Candida krusei*. Slide (S1): Growth control; (S2): Effect of essential oil at a concentration of 512 µg/mL; (S3): Effect of essential oil at a concentration of 256 µg/mL; (S4): Effect of fluconazole at a concentration of 512 µg/mL; (S5): Effect of fluconazole at a concentration of 256 µg/mL. Display 400× enlarged.

For the *C. krusei* strain, fluconazole decreased virulence at the two concentrations evaluated (512 µg/mL and 256 µg/mL) (Figure 5, S4 and S5). At a concentration of 512 µg/mL, the essential oil of *A. confertus* also inhibited the growth of *C. krusei* (S2) hyphae similar to the positive control fluconazole; however, when evaluated at a concentration of 256 µg/mL, the oil did not show an effect on the virulence of the fungal strain (S3), presenting cytoplasmic projections similar to the growth control.

**Figure 6:**
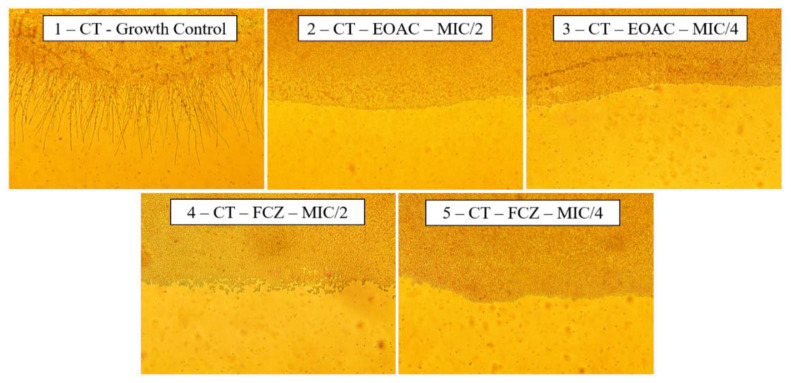
Effects of *Acritopappus confertus* essential oil on the morphological aspects of *Candida tropicalis*. Slide (S1): Growth control; (S2): Effect of essential oil at a concentration of 1024 µg/mL; (S3): Effect of essential oil at a concentration of 512 µg/mL; (S4): Effect of fluconazole at a concentration of 1024 µg/mL; (S5): Effect of fluconazole at a concentration of 512 µg/mL. Display 400× enlarged.

The *C. tropicalis* strain was sensitive to all treatments analyzed, with no observable growth of hyphae in the concentrations of 1024 µg/mL and 512 µg/mL of *A. confertus* essential oil (S2, S3), nor in the positive control fluconazole at concentrations of 1024 µg/mL and 512 µg/mL (S4, S5) (Figure 6). In the growth control (S1) using only nutrient-depleted culture medium, it was possible to observe the projections of hyphae and pseudohyphae of *C. tropicalis*.

### 2.4. Antibacterial and Antibiotic Modifying Activity

The antibacterial activity of the *A. confertus* essential oil was determined by the minimum inhibitory concentration (MIC) according to the results described in Table 3. It was verified that the *A. confertus* oil did not present direct antibacterial activity against most of the standard and multidrug-resistant strains tested, presenting a MIC above 512 μg/mL. However, an exception was observed against the standard strain *S. aureus* ATCC 25923, where the oil had a MIC of 256 μg/mL. This concentration is indicative of clinical relevance and intrinsic antibacterial activity.

**Figure 7:**
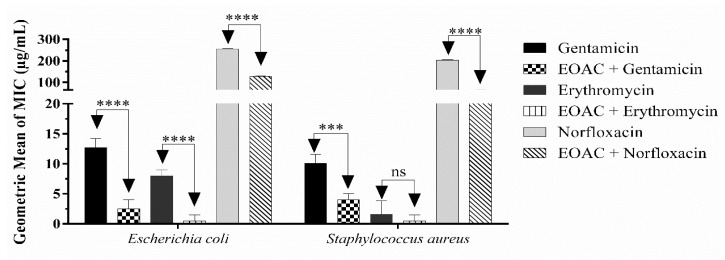
Modifying effect of *Acritopappus confertus* essential oil (EOAC) against the multidrug-resistant strains *Staphylococcus aureus* 10 and *Escherichia coli* 06, in association with the antibiotics gentamicin, erythromycin, and norfloxacin in μg/mL. Statistically significant value with *** *p >* 0.001 and **** *p* < 0.0001.

When tested at sub-inhibitory concentrations (MIC/8) in association with standard antibiotics, the essential oil of *A. confertus* demonstrated a synergistic effect, potentiating the action of the drugs against the multidrug-resistant strains tested (Figure 7). Against *E. coli* 06, the oil reduced the MIC of gentamicin (12.69 to 2.51 μg/mL), erythromycin (8 to 0.5 μg/mL), and norfloxacin (256 to 128 μg/mL). Against *S. aureus* 10, it reduced the MIC of gentamicin (10.07 to 4 μg/mL) and norfloxacin (203.18 to 54 μg/mL), with no modifying effect only in the combination of essential oil with erythromycin. These promising results indicate that *A. confertus* oil may act by increasing the susceptibility of resistant strains against synthetic drugs.

## 3. Discussion

The emergence of fungal strains resistant to standard drugs has grown in recent decades, especially nosocomial ones, such as *Candida* spp. An aggravating factor in this problem is that the development of new drugs has not followed this evolution, which results in a constant struggle against the microscopic world. In this study, it was possible to show that the essential oil of a medicinal species, *A. confertus*, has an anti-*Candida* effect, in addition to being able to intensify the action of fluconazole.

As well as the results found in the present work on the phytochemical composition, Lima et al. [28] reported that the essential oil of *A. confertus* leaves has a predominance of monoterpenes, corresponding to about 81.0% of the total compounds. In addition, the authors identified myrcene (52.0%), β-pinene (16.8%), and limonene (8.2%) as major constituents. Similar results were also observed by Sousa et al. [29], where the main phytochemicals identified in the essential oil of *A. confertus* leaves were myrcene (49.16%), *β*-pinene (17.09%), and limonene (8.73%). The monoterpene myrcene stands out for being one of the major compounds in the essential oil of other species of the Asteraceae family, including *Cladanthus arabicus* Cass. [30], *Pteronia incana* (Burm.) DC. [31,32], *Pteronia onobromoides* DC. [33], *Glebionis coronaria* (L.) Tzvelev [34], *Vernonia polyanthes* Less [35], *Coreopsis triloba* SF Blake [36], and *Montanoa quadrangularis* Schultz Bipontinus [37].

In a study carried out by Tavares et al. [38], the isolated myrcene compound was found to have an antifungal activity against the strains *Candida albicans* ATCC 10231 (MIC: 5 µg/mL), *Candida tropicalis* ATCC 13803 (MIC: 20 µg/mL), and *Candida krusei* H9 (MIC: 10 µg/mL). Negative results were observed by Taweechaisupapong et al. [39], where myrcene was found to be inactive against *C. albicans* and *C. krusei* at the concentrations tested (0.06–32 μL/mL). According to Donati et al. [40], myrcene was evaluated against *Candida parapsilosis* and showed an inhibition zone diameter of only 8.3 ± 0.3 mm when compared to the fluconazole control (18.3 ± 0.3 mm) by the disk diffusion method. Jain and Sharma [41] reported that myrcene had a moderate effect against *C. albicans* with a MIC value of <2.1 μL/mL. Given these data, it is suggested that myrcene, the major compound of the essential oil of *A. confertus*, may be related to the anti-*Candida* activity evaluated in this study, but it cannot be considered as a compound that presents excellent antifungal results.

The compounds *β*-pinene and limonene, identified in reasonable concentrations in the essential oil of *A. confertus*, were also evaluated by other authors for their anti-*Candida* potential [42,43,44,45,46]. According to Rajput and Karuppayil [43], the monoterpene *β*-pinene had a MIC of 1 mg/mL against *C. albicans*. This effect was considered moderate by the authors. Prabajati et al. [47] reported that the mechanism of action of *β*-pinene may be involved in the disruption of the cell membrane of *C. albicans* or by interfering with the functioning of mitochondria. In a study carried out by Thakre et al. [45], it was observed that limonene stood out as a fungicidal compound inhibiting 99.9% of the growth of 35 clinical isolates and 2 standard strains of *C. albicans* under a concentration of 20 mM. These authors also reported that the mechanism of action of limonene may be involved in the damage caused to the integrity of the yeast membrane. Muñoz et al. [46] reported that there was a significant inhibition in the cell viability of *C. albicans* when the strains were previously treated with the compound limonene, at concentrations of 500 μM and 600 μM, for 8 h.

Reports on the antifungal potential of the essential oil of *A. confertus* were not found in the literature; however, other species of Asteraceae have shown promising results as fungicides [48,49,50,51]. The essential oil of *Calendula officinalis* L., for example, showed a high anti-*Candida* activity by the disk diffusion method with inhibition zones of 20–27 mm, against *C. albicans*, *C. dubliniensis* ATCC 777, *C. parapsilosis* ATCC 22019, *C. tropicalis*, *C. guilliermondii,* and *C. glabrata* [48]. According to Silverio et al. [50], essential oils extracted from *Eremanthus erythropappus* (DC) McLeisch were effective against *C. albicans* and *C. tropicalis*, inhibiting fungal growth between 15 and 125 μg/mL. As in the present study, Rodrigues et al. [51] also reported that when associated with the antifungal fluconazole, the essential oil of *Vanillosmopsis arborea* Barker showed modulatory potential and synergistic activity against different strains of *Candida* at lower sub-inhibitory concentrations than the commercial drug tested alone.

Essential oils from the Asteraceae family have also been evaluated for their potential in relation to fungal virulence [52,53]. Stojanović-Radić et al. [52] reported that in addition to its effect on cell growth, *Inula helenium* L. essential oil also significantly decreases *Candida* spp. virulence factors, including the inhibition of biofilm, germ tubes, and phospholipase production. According to Silva et al. [53], the essential oil of *Baccharis trimera* (Less.) DC significantly inhibited the morphological changes associated with the increased virulence and pathogenicity of strains of *C. albicans* (CAINCQS 40006), *C. tropicalis* (CT INCQS 40042), and *C. krusei* (CK INCQS 40095). These data corroborate the results found in the present study, where the essential oil of *A. confertus* inhibited the growth of hyphae of *C. albicans*, *C. krusei,* and *C. tropicalis*, being the first work to evidence the antipleomorphic effect of this species.

The combination of fluconazole with *A. confertus* essential oil may be an alternative to circumvent fungal resistance, wherein some resistant strains are able to expel the drug from the cytoplasmic contents by means of efflux pumps. As fluconazole inhibits the synthesis of ergosterol by inhibiting the 14α-demethylase enzyme that is present in the cell membrane, its action will only occur with its presence in the intracellular environment. Therefore, an inhibition of the efflux pump genes is necessary, which may be the possible targets of the essential oil under study [54].

Regarding antibacterial activities, the *A. confertus* species is still poorly investigated. Fernandes et al. [55] tested the essential oil of *A. confertus* leaves against the bacteria *Escherichia coli* and *Bacillus subtilis* in diffusion assays in solid media and assays in liquid media, with concentrations ranging from 1000 to 4000 ppm. It was verified that the oil of *A. confertus* presented only a bacteriostatic effect against *B. subtilis* and did not demonstrate any effect against *Escherichia coli* by any of the methods used. As these data are preliminary, further studies are needed to verify whether the species lacks direct antibacterial activity. However, it should be noted that products without isolated antibacterial activity are less susceptible to acquiring resistance when used as modifiers of antibiotic action, consequently presenting more potential to combat resistant bacteria by the use of combinations [56].

The terpene myrcene, the major compound in the essential oil of *A. confertus*, in a study by Inoue et al. [57], inhibited the growth of *Staphylococcus aureus* and caused damage to cell membranes by the leakage of K^+^ ions, either alone or in combination with the compound terpinen-4-ol. In association with penicillin in a study by Galluci et al. [58], the interaction of myrcene with the antibiotic showed a negligible clinical effect against *S. aureus* and antagonism against *E. coli*. However, when combined with antibiotics against the tuberculosis-causing bacterium *Mycobacterium tuberculosis*, myrcene was able to decrease the MIC from 16 to 3.9 μg/mL (ethambutol), from 32 to 0.95 μg/mL (isoniazid), and from 16 to 0.475 μg/mL (rifampicin) [59], thus suggesting that the synergistic effect of this compound varies according to the class of antibiotics and types of strains tested.

Other terpenes present in the essential oil of *A. confertus* have a great antibacterial potential according to the literature. The *β*-pinene compound studied by Leite et al. [60] demonstrated activity against the Gram-positive bacteria *Staphylococcus aureus*, *S. epidermidis*, *Streptococcus pneumoniae,* and *S. pyogenes*. The MIC of *β*-pinene ranged from 20 to 40 μg/mL, significantly inhibiting the growth and cell viability of the strains. On the other hand, the monoterpene limonene during in vitro and in silico tests decreased the MIC of the ethidium bromide positive control against the efflux pump mechanism-carrying *S. aureus* strain 1119B. It also augmented the action of norfloxacin against the strain, indicating its potential as a possible inhibitor of the NorA pump that expels hydrophilic fluoroquinolones [61].

While the molecular mechanisms involved in the action of essential oils on bacterial cells are not fully elucidated, it is suggested that due to their lipophilicity, terpenoid compounds may alter membrane permeability, interfering with the transport of a wide variety of substances, thus facilitating the entry of antibiotics into the cytoplasm [62]. However, the sensitivity of a strain to an essential oil varies depending on the complexity of its constituents and the resistance mechanisms of microorganisms. The modifying effect of *A. confertus* observed in this study may be related to the ability of essential oils to interact with the plasma membrane, disrupting amphiphilic peptides and causing disruption of the cytoplasm and damage to lipids and proteins [63,64,65].

## 4. Materials and Methods

### 4.1. Collection of Botanical Material

Fresh leaves of *Acritopappus confertus* were collected in an area of Chapada do Araripe (−7°28′91.81″ S and −39°54′50.33″ W) in the municipality of Crato, Ceará, Brazil. Flowering branches were also collected, pressed, and dehydrated to make an exsiccate of the material deposited and identified in the Herbarium Caririense Dárdano de Andrade-Lima (HCDAL) of the Regional University of Cariri (voucher: 15.148). All botanical material was registered in the National System for the Management of Genetic Heritage and Associated Traditional Knowledge of Brazil (SisGen) (A0B4402), and in the System of Authorization and Information on Biodiversity (SISBIO) of ICMBio (82789-1). 

### 4.2. Essential Oil Extraction

The essential oil extraction followed the methodology of Matos [66] with some modifications. In a hydrodistillation system, 200 g of each fresh plant sample was placed in a glass flask with 2 L of distilled water, and heated to boiling for a period of 2 h. After obtaining the water/oil mixture in the Clevenger type doser, the oil was separated and collected. After extraction, the essential oil of *A. confertus* was dried with anhydrous sodium sulfate (Na_2_SO_4_), presenting a yield of 0.36%.

### 4.3. Analysis of the Essential Oil’s Chemical Composition

#### Essential Oil Analysis

Volatile constituents were analyzed by a gas chromatograph (Agilent Technologies System 6890N GC-FID, Santa Clara, CA, USA) equipped with a DB-5 capillary column (30 m × 0.25 mm, 0.25 µm) coupled to a flame ionization detector (FID). The initial temperature of the inlet and detector were set to 280 °C. Helium gas was used as the mobile phase at a flow rate of 1.0 mL/min. The thermal programmer consisted of an initial temperature of 50 °C, reaching a temperature of 300 °C at a rate of 5 °C/min, totaling 50 min. A total of 1 μL of the oil was injected into the column, diluted in chloroform at a ratio of 1:10 *v/v*. The relative concentrations of the constituents were calculated based on the areas of the GC peaks without correction factors. Qualitative analysis was performed on an Agilent Technologies AutoSystem XL GC/MS device operating in EI mode at 70 eV, equipped with a split/splitless injector (220 °C), with an initial temperature of 280 °C. Columns with the following specifications were used: HP 5MS (30 m × 0.25 mm, 0.25 µm) and HP Innowax (30 m × 0.32 mm, 0.50 µm). Helium was used as the carrier gas at a transport rate of 1.0 mL/min. The thermal programmer consisted of an initial temperature of 50 °C which reached a temperature of 300 °C at a rate of 5 °C/min. A total of 1 μL of the oil was injected into the column, diluted in chloroform (1:10 *v/v*). The retention index was obtained by injecting a mixture of hydrocarbons (C7-C30) under the same conditions as the samples. After chromatographic analysis, the identification of the phytoconstituents of the essential oil of *A. confertus* was carried out based on the retention indexes (RI), determined with reference data from a homologous series of n-alkanes, C7-C30, under identical experimental conditions. The relative amounts of individual components were calculated based on GC peak areas (FID response). After, we compare with the mass spectra library (NIST and Wiley) and the spectral literature according to Adams [67]. 

### 4.4. Antifungal Activity

#### 4.4.1. Fungal Strains

For antifungal testing of the essential oil of *A. confertus*, standard strains of *Candida* were used, acquired from the Oswaldo Cruz Culture Collection of the National Institute for Quality Control in Health (INCQS): *Candida albicans*—CA INCQS 90028, *Candida krusei*—CK INCQS 40095, and *Candida tropicalis*—CT INCQS 40042.

#### 4.4.2. Fungal Culture

All fungal strains were initially cultivated in Sabouraud dextrose agar (SDA) culture medium (37 °C, 24 h) and in doubly concentrated Sabouraud dextrose broth (SDB) medium during the experiments. The depleted potato dextrose agar medium was used to stimulate the projection of hyphae and pseudohyphae in the morphological alteration verification test. To prepare the inoculum, small aliquots of the strains were transferred to tubes containing saline solution (0.9% NaCl) and compared to the McFarland scale, resulting in a concentration of 1 × 10^5^ cells/mL. The essential oil and fluconazole (150 mg, Gn-Germed, Campinas, SP, BR) were measured (150 mg) on an analytical balance and diluted in 1 mL of dimethylsulfoxide (DMSO, Vetec, Duque de Caxias, RJ, BR) and sterile distilled water, respectively, until a matrix concentration of 2048 µg/mL was obtained.

#### 4.4.3. Determination of IC_50_ and Cell Viability Curve

To carry out this experiment, the broth microdilution method according to Javadpour et al. [68] was used, with some modifications. Initially, 96-well plates were filled with 90 μL of SDB and then microdiluted to the penultimate well with 100 μL of the essential oil, obtaining concentrations between 1024 and 1 µg/mL. After this process, 10 µL of fungal inoculum was added. The last well was not microdiluted and was used for growth control. Sterility controls on the culture medium and substance dilution controls were also performed. The plates were incubated at 37 °C for 24 h, and subsequently, the reading was performed in an ELISA spectrophotometer at a wavelength of 630 nm. All experiments were performed in triplicate, and the data obtained were analyzed to construct the cell viability curve and calculate the IC_50_ of the essential oil of *A. confertus* and the reference antifungal (fluconazole). 

#### 4.4.4. Assessment of Fluconazole Modifying Activity

After defining the minimum inhibitory concentration (MIC), the essential oil was tested at sub-inhibitory concentrations (MIC/8) [68]. The 96-well plates were filled with a solution of CSD medium and essential oil at a sub-inhibitory concentration, then 100 μL of fluconazole was added to the first well, followed by a serial microdilution in the proportion of 1:1 until the penultimate well; the last well of the plate was considered to represent a growth control. Finally, 10 µL of the fungal inoculum was added to each well. Controls of the sterility of the culture medium and controls of fluconazole dilution were also performed. Finally, the plates were incubated at 37 °C for 24 h. The reading was performed in an ELISA spectrophotometer (wavelength 630 nm). 

#### 4.4.5. Evaluation of Fungal Virulence Inhibition

To verify whether the essential oil of *A. confertus* causes any alterations in fungal morphology by inhibiting the emission of hyphae, sterile micromorphological chambers were set up. In the humid chamber, 3 mL of depleted PDA medium containing the essential oil in sub-inhibitory concentrations (MIC/2 and MIC/4) was poured onto a sterile slide. Aliquots of the fungal inoculum were used to make two parallel streaks on the slides, and then these were covered with a sterile coverslip. The chamber was incubated at 37 °C for 24 h, and after this period, the culture was analyzed under optical microscopy using a 400× magnification. A control for yeast growth was performed, as well as a control with fluconazole for comparison [69].

### 4.5. Antibacterial Activity

#### 4.5.1. Bacterial Strains

For the antibacterial tests, the standard strains were used: *Staphylococcus aureus* ATCC 25923 and *Escherichia coli* ATCC 25922, and the multidrug-resistant clinical isolates: *S. aureus* 10 (straight swab) and *E. coli* 06 (urine).

#### 4.5.2. Bacterial Culture

All bacterial strains were maintained in Heart Infusion Agar (HIA) medium (37 °C, 24 h) and cultured in Brain Heart Infusion (BHI) 10% during the experiments. Cell suspensions were prepared in test tubes containing saline solution, and their turbidity was compared and adjusted with the 0.5 MacFarland scale. For the analysis of antibacterial activity, the essential oil was measured on an analytical balance (10 mg), and later diluted in 1 mL of DMSO to a final concentration of 1024 μg/mL. The antibiotics gentamicin, erythromycin, and norfloxacin were diluted in 1 mL of sterile distilled water and prepared at the same concentration as the oil.

#### 4.5.3. Determination of the Minimum Inhibitory Concentration (MIC)

The MIC was determined by the broth microdilution method. ELISA plates was filled with 100 μL of solution containing 900 μL of 10% BHI and 100 μL of bacterial suspension. Then, serial microdilution (1:1) was performed with 100 μL of *A. confertus* essential oil until the penultimate well of the plate, with final concentrations between 512 and 8 μg/mL. Bacterial growth controls and sterility controls of the culture medium were performed. Subsequently, the plates were left to incubate at 37 °C (24 h). To read the assays, sodium resazurin (Sigma-Aldrich) was used as a colorimetric indicator of bacterial growth by oxidation–reduction. For this purpose, 20 μL of resazurin was added to each well and, after 1 h, the plates were visually analyzed [70].

#### 4.5.4. Antibiotic Modifying Effect Test

To assess the modifying potential of antibiotics, the essential oil was tested at a sub-inhibitory concentration [68]. Initially, solutions containing 10% BHI medium, oil at sub-inhibitory concentration (MIC/8), and 150 μL of inoculum solution were prepared in Eppendorf tubes. Afterwards, the plates were filled with 100 μL of the solution in each well, followed by serial microdilution (ratio of 1:1) with 100 μL of the reference drug (gentamicin, erythromycin, and norfloxacin); the antibiotic concentrations ranged from 512 μg/mL to 1 µg/mL. The plates were incubated at 37 °C (24 h), and then the test reading was performed with the addition of resazurin.

### 4.6. Statistical Analysis

All assays were performed in triplicate, and their means were calculated with their respective standard deviations (±SD). For antimicrobial activities, the means were submitted to a two-way analysis of variance (concentration × species) followed by the Tukey test, being considered significant when *p* < 0.0001. IC_50_ values were obtained by non-linear regression, and the results were calculated from a standard curve. Statistical analysis and graphing was performed in GraphPad Prism version 6.0. 

## 5. Conclusions

We conclude in this study that essential oil obtained from the leaves of *A. confertus* is able to reduce the growth of *Candida* spp., as assessed by the evaluation of the minimum inhibitory concentration. This result has important implications for the human organism, as such microorganisms are part of its microbiota. In addition, the essential oil was able to inhibit the formation of an important fungal virulence factor, the formation of hyphae and pseudohyphae in the yeasts under study, at concentrations of clinical relevance. When tested on bacteria, this oil—rich in the monoterpene myrcene—was able to modify the action of all antibiotics tested, making them more effective against resistant strains when used in combination. These data are promising, but more research is needed to elucidate the mechanisms of action and interaction between essential oil compounds and antimicrobial drugs. 

## Figures and Tables

**Table 1:** *Acritopappus confertus* (Gardner) R. M. King & H. Rob. essential oil constituents.

Compounds	RI ^a^	RI ^b^	Essential Oil
α-Pinene	937	935	3.24
Sabinene	978	978	0.19
β-Pinene	983	981	18.2
Myrcene	987	989	54.71
Limonene	1029	1031	6.52
β-Cubebene	1390	1391	4.81
β-Caryophyllene	1418	1418	1.93
α-Humulene	1454	1459	0.80
α-Eudesmol	1631	1630	0.21
β-Eudesmol	1652	1652	5.72
Total Identified (%)			96.33

Source: Research Data. ^a^ Experimental retention index (based on n-alkane C7-C30 homologous series). ^b^ Literature retention index (Adams, 2017).

**Table 2:** IC_50_ of the essential oil of *Acritopappus confertus* (EOAC) and fluconazole (FCZ) against *Candida albicans*, *Candida krusei,* and *Candida tropicalis*.

Products Tested	*Candida albicans*	*Candida krusei*	*Candida tropicalis*
FCZ	12.33 μg/mL	131.6 μg/mL	362.9 μg/mL
EOAC	22.19 μg/mL	592.6 μg/mL	615.4 μg/mL
FCZ + EOAC	6.53 μg/mL	295.8 μg/mL	2.25 μg/mL

**Table 3:** Minimum inhibitory concentration of *Acritopappus confertus* essential oil against bacterial strains.

Strain	EOAC
*Staphylococcus aureus* ATCC 25923	256 μg/mL
*Staphylococcus aureus* 10	≥512 μg/mL
*Escherichia coli* ATCC 25922	≥512 μg/mL
*Escherichia coli* 06	≥512 μg/mL

## Data Availability

Data is contained within the article.

## References

[1] El-Baky R.M.A., Masoud S.M., Mohamed D.S., Waly N.G., Shafik E.A., Mohamed D.A., Elkady A., Elbadr M.M., Hetta H.F. (2020). Prevalence and some possible mechanisms of colistin resistance among multidrug-resistant and extensively drug-resisant *Pseudomonas aeruginosa* infect. Drug Resist..

[2] Serwecińska L. (2020). Antimicrobials and antibiotic-resistant bacteria: A risk to the environment and to public health. Water.

[3] Amarasiri M., Sano D., Suzuki S. (2020). Understanding human health risks caused by antibiotic resistant bacteria (ARB) and antibiotic resistance genes (ARG) in water environments: Current knowledge and questions to be answered. Crit. Rev. Environ. Sci. Technol..

[4] Vieira A.J.H., Santos J.I. (2017). Mechanisms of *Candida albicans* resistance to the antifungals amphotericin B, fluconazole and caspofungin. RBAC.

[5] Ellah N.H.A., Abdel-Aleem J.A., Abdo M.N., Abou-Ghadir O.F., Zahran K.M., Hetta H.F. (2019). Efficacy of ketoconazole gel-flakes in the treatment of vaginal candidiasis: Formulation, in vitro and clinical evaluation. Int. J. Pharm..

[6] Khan A., Miller W.R., Arias C.A. (2018). Mechanisms of antimicrobial resistance among hospital-associated pathogens. Expert Rev. Anti. Infect. Ther..

[7] Kadri S.S. (2020). Key takeaways from the US CDC’s 2019 antibiotic resistance threats report for frontline providers. Crit. Care Med..

[8] Denamur E., Clermont O., Bonacorsi S., Gordon D. (2021). The population genetics of pathogenic *Escherichia coli*. Nat. Rev. Microbiol..

[9] Ford C.A., Hurford I.M., Cassat J.E. (2021). Antivirulence strategies for the treatment of *Staphylococcus aureus* infections: A mini review. Microbiol. Front..

[10] Guo Y., Song G., Sun M., Wang J., Wang Y. (2020). Prevalence and therapies of antibiotic-resistance in *Staphylococcus aureus*. Front. Cell Infect. Microbiol..

[11] Kumar A., Khan F., Saikia D. (2019). Exploration of Medicinal Plants as Sources of Novel Anticandidal Drugs. Curr. Top. Med. Chem.

[12] Talapko J., Juzbašić M., Matijević T., Pustijanac E., Bekić S., Kotris I., Škrlec I. (2021). *Candida albicans*—The virulence factors and clinical manifestations of infection. J. Fungi.

[13] Navarro-Arias M.J., Hernández-Chávez M.J., Garcia-Carnero L.C., Amezcua-Hernández D.G., Lozoya-Pérez N.E., Martínez-Duncker I., Franco B., Mora-Montes H.M. (2019). Differential recognition of *Candida tropicalis*, *Candida guilliermondii*, *Candida krusei*, and *Candida auris* by human innate immune cells. Infect. Drug Resist..

[14] Kołaczkowska A., Kołaczkowski M. (2016). Drug resistance mechanisms and their regulation in non-*albicans Candida* species. J. Antimicrob. Chemother..

[15] Cid-Chevecich C., Müller-Sepúlveda A., Jara J.A., López-Muñoz R., Santander R., Budini M., Escobar A., Quijada R., Criollo A., Díaz-Dosque M. (2022). *Origanum vulgare* L. essential oil inhibits virulence patterns of *Candida* spp. and potentiates the effects of fluconazole and nystatin in vitro. BMC Complement. Med. Ther..

[16] Sánchez-Martínez C., Pérez-Martín J. (2001). Dimorphism in fungal pathogens: *Candida albicans* and *Ustilago maydis*—Similar inputs, different outputs. Curr. Opin. Microbiol..

[17] McCall A.D., Pathirana R.U., Prabhakar A., Cullen P.J., Edgerton M. (2019). *Candida albicans* biofilm development is governed by cooperative attachment and adhesion maintenance proteins. NPJ Biofilms Microbiomes.

[18] Sharma J., Rosiana S., Razzaq I., Shapiro R.S. (2019). Linking cellular morphogenesis with antifungal treatment and susceptibility in candida pathogens. J. Fungi.

[19] Khan M.S.A., Alshehrei F., Al-Ghamdi S.B., Bamaga M.A., Al-Thubiani A.S., Alam M.Z. (2020). Virulence and biofilms as promising targets in developing antipathogenic drugs against candidiasis. Future Sci. OA..

[20] Nidhi P., Rolta R., Kumar V., Dev K., Sourirajan A. (2020). Synergistic potential of *Citrus aurantium* L. essential oil with antibiotics against *Candida albicans*. J. Ethnopharmacol..

[21] Braga A.L., Cruz R.P., Carneiro J.N.P., Santos A.T.L., Sales D.L., Bezerra C.F., Fonseca V.J.A., Rocha J.E., Freitas T.S., Campina F.F. (2021). Piper regnellii (Miq.) C. DC.: Chemical composition, antimicrobial effects, and modulation of antimicrobial resistance. S. Afr. J. Bot..

[22] Linkoln A., Leal A.L.A.B., Bezerra C.F., Rocha J.E., Santos A.T.L., Cruz R.P., Carneiro J.N.P., Sales D.L., Freitas T.S., Tintino S.R. (2019). *Piper cernuum* Vell.: Chemical profile and antimicrobial potential evaluation. Ind. Crops Prod..

[23] Córdoba S., Vivot W., Szusz W., Albo G. (2019). Antifungal activity of essential oils against *Candida* species isolated from clinical samples. Mycopathologia.

[24] Baj T., Biernasiuk A., Wróbel R., Malm A. (2020). Chemical composition and in vitro activity of *Origanum vulgare* L. *Satureja hortensis* L. *Thymus serpyllum* L. and *Thymus vulgaris* L. essential oils towards oral isolates of *Candida albicans* and *Candida glabrata*. Open Chem. J..

[25] Salkar K., Suthar A., Chauhan V., Naik V. (2013). Evaluation of selected medicinal plants for anticandida potential. Res. J. Pharm. Biol. Chem. Sci..

[26] Ferronatto R., Marchesan E.D., Pezenti E., Bednarski F., Onofre S.B. (2007). Atividade antimicrobiana de óleos essenciais produzidos por *Baccharis dracunculifolia* D.C. e *Baccharis uncinella* D.C. (Asteraceae). Rev. Bras. Farmacogn..

[27] Funch L.S., Hayley R., Funch R., Giuletti A.M., Melo E. (2004). Chapadada Diamantina a Useful Plants.

[28] Lima M.A.S., Barros M.C.P., Pinheiro S.M., Nascimento R.F., Abreu Matos F.J., Silveira E.R. (2005). Volatile compositions of two asteraceae from the north-east of Brazil: *Ageratum conyzoides* and *Acritopappus confertus* (Eupatorieae). Flavour Fragr. J..

[29] Sousa J.D., Leite T.R., Linhares K.V., Sousa J.D., Bezerra J.W.A., Santos M.A.F., Torquato I.H.S., Boligon A.A., Bezerra J.S., Campos N.B. (2020). Chromatographic profile and allelopathic potential of the essential oil of *Acritopappus confertus* (Gardner) RM King & H. Rob. (Asteraceae). Res. Soc. Dev..

[30] Hanbali F.E., Mellouki F., Akssira M., El Hassani B., Blázquez M.A., Boira H. (2005). Composition and antibacterial activity of essential oils of *Cladanthus arabicus* Cass. (Asteraceae). J. Essent. Oil-Bear. Plants.

[31] Mayekiso B., Magwa M.L., Coopoosamy R.M. (2006). Variation in the essential oil constituents of *Pteronia incana* (Asteraceae). Afr. J. Biotechnol..

[32] Hulley I.M., Viljoen A.M., Tilney P.M., Van Vuuren S.F., Kamatou G.P.P., Van Wyk B.E. (2010). The ethnobotany, leaf anatomy, essential oil variation and biological activity of *Pteronia incana* (Asteraceae). S. Afr. J. Bot..

[33] Hulley I.M., Viljoen A.M., Tilney P.M., Vuuren S.F.V., Kamatou G.P.P., Van Wyk B.E. (2010). Ethnobotany, leaf anatomy, essential oil composition and antibacterial activity of *Pteronia onobromoides* (Asteraceae). S. Afr. J. Bot..

[34] Haouas D., Cioni P.L., Flamini G., Halima-Kamel M.B., Hamouda M.H.B. (2016). Variation of chemical composition in flowers and leaves essential oils among natural population of Tunisian *Glebionis coronaria* (L.) Tzvelev (Asteraceae). Chem. Biodivers..

[35] Moreira R.R.D., Martins G.Z., Varandas R., Cogo J., Perego C.H., Roncoli G., Sousa M.C., Nakamura C.V., Salgueiro L., Cavaleiro C. (2017). Composition and leishmanicidal activity of the essential oil of *Vernonia polyanthes* Less (Asteraceae). Nat. Prod. Res..

[36] Espinosa S., Bec N., Larroque C., Ramírez J., Sgorbini B., Bicchi C., Gilardoni G. (2019). Chemical, enantioselective, and sensory analysis of a cholinesterase inhibitor essential oil from *Coreopsis triloba* SF Blake (Asteraceae). Plants.

[37] Rojas J., Ntoutoume G.M.A.N., Martin P., Morillo M. (2021). Antibacterial Activity and Reversal of Multidrug Resistance of Tumor Cells by Essential Oils from Fresh Leaves, Flowers, and Stems of *Montanoa quadrangularis* Schultz Bipontinus (Asteraceae) Collected in Mérida—Venezuela. Biomolecules.

[38] Tavares A.C., Gonçalves M.J., Cruz M.T., Cavaleiro C., Lopes M.C., Canhoto J., Salgueiro L.R. (2010). Essential oils from *Distichoselinum tenuifolium*: Chemical composition, cytotoxicity, antifungal and anti-inflammatory properties. J. Ethnopharmacol..

[39] Taweechaisupapong S., Aieamsaard J., Chitropas P., Khunkitti W. (2012). Inhibitory effect of lemongrass oil and its major constituents on *Candida* biofilm and germ tube formation. S. Afr. J. Bot..

[40] Donati M., Mondin A., Chen Z., Miranda F.M., Nascimento B.B., Schirato G., Pastore P., Froldi G. (2014). Radical scavenging and antimicrobial activities of *Croton zehntneri*, *Pterodon emarginatus* and *Schinopsis brasiliensis* essential oils and their major constituents: Estragole, trans-anethole, β-caryophyllene and myrcene. Nat. Prod. Res..

[41] Jain N., Sharma M. (2020). Inhibitory effect of some selected essential oil terpenes on fungi causing superficial infection in human beings. J. Essent. Oil Bear. Plants.

[42] Silva A.C.R.D., Lopes P.M., Azevedo M.M.B.D., Costa D.C.M., Alviano C.S., Alviano D.S. (2012). Biological activities of α-pinene and β-pinene enantiomers. Molecules.

[43] Rajput S.B., Karuppayil S.M. (2013). Small molecules inhibit growth, viability and ergosterol biosynthesis in *Candida albicans*. Springerplus.

[44] Andrade A.C.M., Rosalen P.L., Freires I.A., Scotti L., Scotti M.T., Aquino S.G., Castro R.D. (2018). Antifungal activity, mode of action, docking prediction and anti-biofilm effects of (+)-β-pinene enantiomers against *Candida* spp.. Curr. Top. Med. Chem..

[45] Thakre A., Zore G., Kodgire S., Kazi R., Mulange S., Patil R., Shelar A., Santhakumari B., Kulkarni M., Kharat K. (2018). Limonene inhibits *Candida albicans* growth by inducing apoptosis. Med. Mycol. J..

[46] Muñoz J.E., Rossi D.C., Jabes D.L., Barbosa D.A., Cunha F.F., Nunes L.R., Arruda D.C., Taborda C.P. (2020). In Vitro and In Vivo Inhibitory Activity of Limonene against Different Isolates of *Candida* spp.. J. Fungi.

[47] Prabajati R., Hernawan I., Hendarti H.T. (2017). Effects of *Citrus limon* essential oil (*Citrus limon* L.) on cytomorphometric changes of *Candida albicans*. DJMKG.

[48] Gazim Z.C., Rezende C.M., Fraga S.R., Svidzinski T.I.E., Cortez D.A.G. (2008). Antifungal activity of the essential oil from *Calendula officinalis* L. (Asteraceae) growing in Brazil. Braz. J. Microbiol..

[49] Zapata B., Duran C., Stashenko E., Betancur-Galvis L., Mesa-Arango A.C. (2010). Antifungal activity, cytotoxicity and composition of essential oils from the Asteraceae plant family. Rev. Iberoam. Micol..

[50] Silvério M.S., Del-Vechio-Vieira G., Pinto M.A., Alves M.S., Sousa O.V. (2013). Chemical composition and biological activities of essential oils of *Eremanthus erythropappus* (DC) McLeisch (Asteraceae). Molecules.

[51] Rodrigues F.F.G., Colares A.V., Nonato C.D.F.A., Galvão-Rodrigues F.F., Mota M.L., Braga M.F.B.M., Costa J.G.M. (2018). In vitro antimicrobial activity of the essential oil from *Vanillosmopsis arborea* Barker (Asteraceae) and its major constituent, α-bisabolol. Micróbio. Pathog..

[52] Stojanović-Radić Z., Dimitrijević M., Genčić M., Pejčić M., Radulović N. (2020). Anticandidal activity of *Inula helenium* root essential oil: Synergistic potential, anti-virulence efficacy and mechanism of action. Ind. Crops Prod..

[53] Silva T.G., Silva J.C.P., Carneiro J.N.P., Amaral W., Deschamps C., Araújo J.P., Costa J.G.M., Almeida W., Silva L.E., Coutinho H.D.M. (2021). Phytochemical characterization and inhibition of *Candida* sp. by the essential oil of *Baccharis trimera* (Less.) DC. Arch. Microbiol..

[54] Keereedach P., Hrimpeng K., Boonbumrung K. (2020). Antifungal activity of Thai cajuput oil and its effect on efflux-pump gene expression in fluconazole-resistant *Candida albicans* clinical isolates. Int. J. Microbiol..

[55] Fernandes E., Sousa M.J., Dias A. (2014). Evaluation of antimicrobial activity of essential oils from the Brazilian plants *Acritopappus confertus*, *Cuphea carthagenensis* and *Poiretia bahiana*. Planta Med..

[56] Bhardwaj A.K., Mohanty P. (2012). Bacterial efflux pumps involved in multidrug resistance and their inhibitors: Rejuvinating the antimicrobial chemotherapy. Recente Pat. Antiinfect. Drug Discov..

[57] Inoue Y., Shiraishi A., Hada T., Hamashima H., Shimada J. (2004). The antibacterial effects of myrcene on *Staphylococcus aureus* and its role in the essential oil of the tea tree (*Melaleuca alternifolia*). Nat. Med..

[58] Gallucci N., Casero C., Oliva M., Zygadlo J., Demo M. (2006). Interaction between terpenes and penicillin on bacterial strains resistant to beta-lactam antibiotics. Mol. Med. Chem..

[59] Sieniawska E., Swatko-Ossor M., Sawicki R., Skalicka-Woźniak K., Ginalska G. (2017). Natural terpenes influence the activity of antibiotics against isolated *Mycobacterium tuberculosis*. Clínica Med. Princ..

[60] Leite A.M., Lima E.D.O., Souza E.L.D., Diniz M.D.F.F.M., Trajano V.N., Medeiros I.A.D. (2007). Inhibitory effect of beta-pinene, alpha-pinene and eugenol on the growth of potential infectious endocarditis causing Gram-positive bacteria. Rev. Bras. Cienc. Farm..

[61] Araújo A.C.J., Freitas P.R., Barbosa C.R.S., Muniz D.F., Almeida R.S., Menezes I.R.A., Ribeiro-Filho J., Tintino S.R., Coutinho H.D.M. (2021). In vitro and in silico inhibition of *Staphylococcus aureus* efflux pump NorA by α-pinene and limonene. Curr. Microbiol..

[62] Freitas P.R., Araújo A.C.J., Barbosa C.R., Muniz D.F., Tintino S.R., Ribeiro-Filho J., Júnior J.P.S., Filho J.M.B., Sousa G.R., Coutinho H.D. (2021). Inhibition of Efflux Pumps by Monoterpene (α-Pinene) and impact on *Staphylococcus aureus* Resistance to Tetracycline and Erythromycin. Curr. Drug Metab..

[63] Azevedo I.L., Almeida A.C., Martins E.R., Nogueira W.C.L., Faria Filho D.E., Oliveira S.P., Prates J.P.B., Souza C.N. (2016). Eficácia in vitro do óleo essencial de capim-limão (cymbopogon flexuosus steud. wats.) frente a bactérias entéricas de origem avícola. Acta Vet. Bras..

[64] Bakkali F., Averbeck S., Averbeck D., Idaomar M. (2008). Biological effects of essential oils--a review. Food Chem. Toxicol..

[65] Mollica A., Macedonio G., Stefanucci A., Costante R., Carradori S., Cataldi V., Giulio M.D., Cellini L., Silvestri R., Giordano C. (2018). Arginine-and lysine-rich peptides: Synthesis, characterization and antimicrobial activity. Lett. Drug Des. Discov..

[66] Matos F.J.A. (1997). Introdução à Fitoquímica Experimental.

[67] Adams R.P. (2007). Identification of Essential Oil Components by Gas Chromatography/Mass Spectroscopy.

[68] Coutinho H.D.M., Costa J.G.M., Lima E.O., Falcão-Silva V.S., Siqueira- Júnior J.P. (2008). Enhancement of the antibiotic activity against a multiresistant *Escherichia coli* by *Mentha arvensis* L. and Chlorpromazine. Chemotherapy.

[69] Sidrim J.J.C., Rocha M.F.G. (2010). Micologia Médica à Luz de Autores Contemporâneos.

[70] Javadpour M.M., Juban M.M., Lo W.C.J., Bishop S.M., Alberty J.B., Cowell S.M., Becker C.L., McLaughlin M.L. (1996). De novo antimicrobial peptides with low mammalian cell toxicity. J. Med. Chem..

